# Rare Manifestation of Sjogren's Syndrome: Renal Tubular Acidosis‐Induced Hypokalemic Paralysis—A Case Report

**DOI:** 10.1002/ccr3.72655

**Published:** 2026-05-03

**Authors:** Premendra Vimal, Dhiraj Chaurasia, Dipesh Jha, Raj Adhikari

**Affiliations:** ^1^ Department of Internal Medicine Kathmandu Medical College Kathmandu Nepal

**Keywords:** autoimmune disorder, case report, hypokalemia, renal tubular acidosis, Sjogren's syndrome

## Abstract

Sjogren's syndrome (SS) is an autoimmune disorder characterized by inflammation of exocrine glands, often presenting with symptoms such as dry eyes and mouth. Although less common, renal involvement can lead to serious complications like hypokalemic paralysis. We report a case of a 28‐year‐old female with no prior medical history, who initially presented with fever, myalgia, and arthralgia. Her condition later progressed to hypokalemia and metabolic acidosis. Further evaluation confirmed Type 1 renal tubular acidosis (RTA) secondary to SS. Treatment with potassium supplementation, steroids, and bicarbonate resolved the symptoms. This case highlights the importance of considering renal involvement in SS patients and underscores the need for prompt intervention to prevent severe complications.

**Key Clinical Message**

Sjögren's syndrome can rarely manifest as hypokalemic paralysis caused by distal renal tubular acidosis (RTA), sometimes before the appearance of classic sicca symptoms. This uncommon and often overlooked presentation may resemble primary neurological disorders. Recognizing renal tubular dysfunction early is crucial to avoid diagnostic delays and prevent life‐threatening complications from severe hypokalemia.

## 1 Introduction

Sjögren's syndrome (SS) is a chronic autoimmune disorder characterized by lymphocytic infiltration of exocrine glands, leading to dryness of the eyes and mouth. Extra‐glandular involvement in SS also occurs, including glomerulonephritis, tubulointerstitial nephritis, arthritis, arthralgia, vasculitis, pulmonary diseases, and lymphomas. The renal involvement in SS typically includes tubulointerstitial nephritis (TIN), causing tubular dysfunction and, less commonly, glomerulonephritis. Distal renal tubular acidosis (RTA) is the most common tubular dysfunction in SS [1, 2]. Hypokalemia and metabolic acidosis in distal RTA cause transient muscle weakness, paralysis, and osteomalacia [3]. Among the many causes of hypokalemia, distal renal tubular acidosis (RTA) due to Sjogren's syndrome is an important entity. Here we report a case referred to our hospital with complaints of generalized weakness with pain in the left lower limb, fatigue, and decreased appetite.

## 2 Case History/Examination

A 28‐year‐old female with no comorbidities was referred and presented to our hospital with complaints of generalized weakness, more pronounced weakness and pain in the left lower limb, fatigue, and decreased appetite. Previously, she was admitted and was being treated in another hospital, where she initially presented with a 14‐day history of low‐grade intermittent fever associated with generalized body ache, generalized weakness, myalgia, arthralgia, headache, and burning micturition. There is also no history of paralysis, double vision, drooping of eyelid, difficulty in speech, swallowing, or regurgitation. On examination, her vitals were a pulse rate of 68 beats per minute, a temperature of 98.0°F, a blood pressure of 120/80 mmHg, and a respiratory rate of 16 breaths per minute. Physical examination showed negative Trousseau and Chvostek signs. On neurological exam, the patient showed bilateral motor weakness, more pronounced proximally. Power was 3/5 in the shoulders and right hip, and 2/5 in the left hip. Distal muscle strength was relatively better preserved. There was no sensory deficit, and the rest of the systemic exam was unremarkable. She was admitted to the high‐care medicine unit for further evaluation.

## 3 Differential Diagnosis, Investigations, and Treatment

Initial laboratory results showed hypokalemia (2.4 mEq/L) and hypocalcemia (6.9 mg/dL). Serum albumin was 3.8 g/dL, and the albumin‐corrected calcium level was 7.06 mg/dL. Hematological parameters were within normal limits, with a total leukocyte count of 9,200/mm^3^ and a platelet count of 157,000/mm. Renal function was preserved, with a serum urea of 24 mg/dL and creatinine of 0.6 mg/dL. Liver transaminases were significantly elevated, with aspartate aminotransferase (AST) at 136 U/L and alanine aminotransferase (ALT) at 244 U/L. Inflammatory markers showed an elevated C‐reactive protein (CRP) of 18 mg/L (Table 1), and serum magnesium was profoundly reduced at 0.5 mg/dL. A mildly elevated lactate level of 2.5 mmol/L was noted (Table 2).

**TABLE 1:** Hematological and biochemical parameters.

Parameter	Result	Reference range
Total leukocyte count	9,200/mm^3^	4,000–11,000
Neutrophils	77%	40%–75%
Lymphocytes	20%	20%–40%
Hemoglobin	10.3 g/dL	12–16 g/dL
Platelets	157,000/mm^3^	150,000–450,000
ALT (SGPT)	244 U/L	< 40 U/L
AST (SGOT)	136 U/L	< 40 U/L
Sodium	131.2 mmol/L	135–145
Potassium	2.4 mEq/L	3.5–5.0
Albumin	3.8 g/dL	3.5–5.0
Calcium	6.9 mg/dL	8.5–10.5
Ionized calcium	0.92 mmol/L	1.12–1.32
Magnesium	0.5 mg/dL	1.7–2.2
Urea	24 mg/dL	10–50
Creatinine	0.6 mg/dL	0.6–1.2
CRP	18 mg/L	< 5
Glucose	152 mg/dL	70–140

**TABLE 2:** Arterial blood gas and urinary electrolytes.

Parameter	Result	Reference range
pH	7.18	7.35–7.45
pCO_2_	34.7 mmHg	35–45
pO_2_	103.5 mmHg	80–100
HCO_3_^−^	12 mmol/L	22–26
Anion gap	10.4 mmol/L	8–12
Lactate	2.5 mmol/L	0.5–2.2
Urine pH	7.0	4.5–8.0
Urine sodium	145.5 mmol/L	—
Urine potassium	7.4 mmol/L	—
Urine chloride	61 mmol/L	—
24‐h urine calcium	520 mg/24 h	< 250 mg/24 h
Urinary phosphorus	11.25 mg/dL	20–60 mg/dL

Arterial blood gas analysis demonstrated acidemia (pH 7.18) with low bicarbonate (12 mmol/L) and pCO_2_ of 34.7 mmHg, along with a normal serum anion gap of 10.4 mmol/L (Table 2), consistent with a normal anion gap (hyperchloremic) metabolic acidosis. The combination of a normal serum anion gap and an elevated urinary anion gap was a strong indicator of distal (Type 1) renal tubular acidosis. Spot urine analysis showed an inappropriately alkaline pH of 7.0 in the setting of systemic metabolic acidosis and a positive urinary anion gap (Na^+^ 145.5 mmol/L, K^+^ 7.4 mmol/L, Cl^−^ 61 mmol/L), indicating impaired distal ammonium excretion. Since the patient had no history suggestive of gastrointestinal potassium loss, a renal tubular defect of probable autoimmune etiology was considered. A 24‐h urine collection demonstrated hypercalciuria (520 mg/24 h) and low urinary phosphorus (11.25 mg/dL). Abdominal ultrasonography was unremarkable, effectively excluding obstructive uropathy. After excluding drug‐induced tubular dysfunction and obstructive causes, an autoimmune etiology was considered probable.

Evaluation for infectious etiologies was negative for hepatitis B surface antigen, anti‐HCV antibodies, and HIV, suggesting no chronic infections contributing to tubular dysfunction. Endocrine causes were excluded based on normal thyroid function tests (FT3 4.17 pg/mL, FT4 1.68 ng/dL, TSH 1.89 μIU/mL) and a normal vitamin D level of 56.1 ng/mL (Table 3), ruling out thyroid dysfunction and vitamin D deficiency as contributors to the metabolic and electrolyte disturbances. Electrocardiography was normal despite severe hypokalemia. The elevated serum glucose level (152 mg/dL) was attributed to stress hyperglycemia.

**TABLE 3:** Autoimmune, infectious, and endocrine profile.

Test	Result	Reference range
ANA	0.6	< 1.0
SS‐A (Ro 60 kDa)	8 RU/mL	< 5
Ro‐52	12 RU/mL	< 20
Anti‐dsDNA	Negative	Negative
FT3	4.17 pg/mL	2.0–4.4
FT4	1.68 ng/dL	0.9–2.3
TSH	1.889 μIU/mL	0.4–4.0
Vitamin D	56.1 ng/mL	30–100
HBsAg	Negative	Negative
Anti‐HCV	Negative	Negative
HIV antibody	Negative	Negative

Ophthalmologic evaluation showed reduced tear production, with Schirmer test results of 3 mm in the left eye and 4 mm in the right eye, and a tear break‐up time of 7 s, supporting an underlying sicca disorder. The ANA screening test was negative (0.6). However, because there was a high index of suspicion for Sjögren's syndrome, a detailed extractable nuclear antigen (ENA) profile was advised. It demonstrated anti‐SSA (Ro60) positivity at 8 RU/mL, while Ro52 was within the laboratory's reference range at 12 RU/mL (Table 3). Although the anti‐SSA titer was low‐positive, even weak SSA reactivity is considered diagnostically meaningful in the presence of objective sicca features and classical extraglandular manifestations, such as distal renal tubular acidosis.

Magnetic resonance imaging (MRI) of the dorsolumbar spine was performed to evaluate the cause of acute weakness and to exclude spinal cord compression or other structural etiologies. It revealed partial disc dehydration of the lumbar intervertebral discs, straightening of the lumbar spine, and a mild diffuse bulge at the L4–L5 level, causing minimal indentation of the anterior thecal sac. These findings were considered incidental and did not account for the sudden onset of weakness. Electrophysiological testing of the left lower limb was performed during the episode of severe hypokalemia. Nerve conduction studies revealed markedly reduced compound muscle action potential (CMAP) amplitudes in the peroneal and tibial nerves, with preserved sensory nerve responses, findings consistent with reversible muscle membrane inexcitability rather than a neuropathic process. Needle electromyography (EMG) demonstrated myopathic motor unit potentials, characterized by short‐duration, low‐amplitude units with early recruitment and no neurogenic features.

Overall, the investigative findings presented a highly suggestive clinical picture: After excluding gastrointestinal, drug‐related, obstructive, infectious, endocrine, and alternative autoimmune causes of distal RTA, the combination of Type 1 RTA, hypercalciuria, objective ocular dryness, and anti‐SSA positivity strongly supported a diagnosis of primary Sjögren's syndrome presenting with distal renal tubular acidosis. The presence of anti‐SSA positivity, together with sicca features and classical distal RTA, further reinforced this diagnosis despite ANA negativity.

## 4 Conclusion and Results (Outcome and Follow‐Up)

The patient's hypokalemia was initially corrected with intravenous potassium chloride administered at 10 mEq/h, delivering a total of 80 mEq over the first 24 h under continuous cardiac monitoring. After stabilization, she was transitioned to oral potassium chloride 40 mEq/day and oral sodium bicarbonate 1 mEq/kg/day to correct the metabolic acidosis associated with distal renal tubular acidosis. She also received pregabalin 75 mg/day and methylcobalamin 1,500 mcg/day for symptomatic relief, and her muscle strength returned to baseline within three days. As the renal tubular dysfunction was attributed to Sjögren's syndrome, she was started on oral methylprednisolone 0.5 mg/kg/day, and hydroxychloroquine 200 mg/day was added as a steroid‐sparing agent. After 14 days of hospitalization, she was discharged on potassium citrate 40 mEq/day and oral sodium bicarbonate for long‐term alkali therapy, along with gabapentin, methylcobalamin, calcium carbonate with vitamin D3, and urinary alkalinizing agents. She remained clinically stable and asymptomatic at her 10‐day follow‐up visit.

Sjögren's syndrome can present with distal renal tubular acidosis and, less commonly, hypokalemic paralysis. Autoimmune etiologies should be considered in patients with unexplained hypokalemia and normal anion gap metabolic acidosis, as timely recognition and treatment may prevent serious complications.

## 5 Discussion

Sjogren's syndrome is a chronic autoimmune exocrinopathy characterized by a slow, progressive course, attributed to monocytic infiltration that leads to secondary invasion of tubular membranes and epithelial linings [2, 4, 5]. Its estimated prevalence is approximately 0.3 to 1 per 1000 persons, with an age incidence peak around 50 years. The condition is more prevalent in females, with a ratio of 9:1 [6, 7, 8]. Significant glandular involvement occurs in exocrine glands such as the lacrimal and salivary glands [5]. Extra‐glandular manifestations of primary Sjogren's syndrome (pSS) encompass renal diseases, pulmonary diseases, hematological diseases, and cardiac diseases, causing prolonged QT intervals [2, 5].

Renal involvement occurs in 16%–67% of patients with primary Sjögren's syndrome (pSS), most commonly presenting as tubulointerstitial nephritis or distal (Type 1) renal tubular acidosis (RTA), which is reported in 4.3%–9% of cases [9]. Renal manifestations may remain subclinical until acute complications such as hypokalemic paralysis, nephrolithiasis, or osteomalacia occur [10]. In this patient, the presence of normal anion gap hyperchloremic metabolic acidosis, inappropriately alkaline urine, and a positive urinary anion gap was diagnostic of Type 1 RTA.

Renal tubular acidosis results from a tubular defect affecting physiological acidification and can stem from congenital causes, nephrotoxic drugs, diuretic overuse, autoimmune disorders, and malignancies. Blood gas analysis in our case revealed normal anion gap hyperchloremic metabolic acidosis, alkaline urine, and hypokalemia, suggestive of Type 1 RTA [2, 5, 11]. Type 1 RTA can result in various ionic and metabolic imbalances, including hypokalemia, hypophosphaturia, and hypocitraturia, with hypokalemia being the most common [12].

Repeated progression of hypokalemia can lead to skeletal muscle potassium release into the extracellular compartment, culminating in hypokalemic paralysis and diminished muscle strength [13, 14]. The exact relationship between Sjögren's syndrome and RTA remains unclear, with proposed mechanisms including a decrease in hydrogen ions due to injury to the H^+^‐ATPase pump of alpha‐intercalated cells and the presence of anti‐carbonic anhydrase autoantibodies [2, 10, 13].

The hypocalcemia in this patient likely resulted from two converging mechanisms: Renal calcium wasting secondary to chronic metabolic acidosis, evidenced by marked hypercalciuria (520 mg/24 h), and impaired PTH‐mediated calcium defense secondary to severe hypomagnesemia (0.5 mg/dL). Although compensatory secondary hyperparathyroidism usually maintains serum calcium within normal limits, chronic metabolic acidosis ultimately leads to a negative calcium balance via renal calcium wasting [15]. Very low magnesium concentrations can paradoxically suppress PTH release via Gα subunit activation, mimicking calcium‐sensing receptor stimulation, and may simultaneously aggravate hypokalemia by disinhibiting renal potassium channels [16]; although PTH levels were not measured in this patient, the serum albumin of 3.8 g/dL confirmed true hypocalcemia (albumin‐corrected calcium 7.06 mg/dL), and magnesium correction was prioritized alongside potassium and bicarbonate replacement. The slightly elevated lactate level (2.5 mmol/L) in the absence of infection was likely due to severe hypokalemia and associated skeletal muscle dysfunction. Significant potassium loss can impair cellular metabolism and contribute to mild, non‐septic lactic acidosis.

During acute hypokalemic periodic paralysis (HypoKPP), low CMAP amplitudes are expected and indicate reversible muscle membrane inexcitability rather than neuropathy, even when focal or asymmetric weakness is present. In our patient, needle EMG showed myopathic motor unit potentials characterized by short‐duration, low‐amplitude units with early recruitment and no neurogenic features, confirming a myopathic rather than axonal process. Sensory studies were normal; CMAP amplitudes improved after potassium correction, and no clinical, laboratory, or electrophysiological evidence of vasculitic neuropathy was found. Importantly, focal or hemiparetic variants of HypoKPP have been described in the literature, demonstrating that localized weakness can occur despite the systemic nature of the disorder [17].

A negative ANA does not exclude the diagnosis of primary Sjögren's syndrome, as ANA negativity is a recognized serological subset of the disease. According to the 2016 ACR/EULAR classification criteria, some patients with primary Sjögren's syndrome may exhibit negative results for antinuclear antibodies, and such patients may differ in their immunological state and extent of organ involvement compared to those who are ANA‐positive [18]. In a cohort of 233 patients fulfilling diagnostic criteria for primary Sjögren's syndrome, ANA‐negative patients were characterized by less frequent use of immunosuppressive therapy, fewer articular manifestations, and a lower disease activity score, suggesting a generally milder systemic phenotype—consistent with the predominantly renal presentation observed in our patient [19].

Though renal biopsy was not performed in our patient to rule out interstitial nephritis, the presence of lacrimal glandular manifestations further prompted the investigation of Sjogren autoantibodies (anti‐Ro/SSA and ‐La/SSB) [2]. Taking into account the 2016 American College of Rheumatology (ACR)/European League Against Rheumatism (EULAR) classification criteria for primary SS, our patient obtained 4 points: 3 points for anti‐SSA/Ro‐positive and 1 point for Schirmer Test ≤ 5 mm [12]. Hereby, the cause for RTA and hypokalemic paralysis is Sjogren syndrome was justified.

Hypokalemia in Sjögren's syndrome can cause severe weakness, ranging from hypokalemic paralysis to quadriparesis and even respiratory failure [2, 4]. This was shown in a recent report by Shaik and Manorenj, where uncorrected hypokalemia resulted in a pseudo–Guillain–Barré syndrome presentation in a patient with Sjögren's syndrome [20]. Prompt identification of the primary cause is essential for effective treatment. Potassium supplementation and steroids played a key role in managing the patient's symptoms, highlighting the importance of comprehensive and timely intervention in addressing serious complications linked to Sjogren's syndrome [4].

## 6 Limitations

This case report has several limitations. First, the 10‐day follow‐up period is short, and repeat electrolyte values, renal function tests, and bicarbonate levels at follow‐up were unavailable at the time of manuscript submission, limiting the assessment of long‐term treatment response. Second, a renal biopsy was not performed to histologically confirm tubulointerstitial nephritis, which would have further strengthened the diagnosis of Sjögren's syndrome‐associated distal RTA. Third, serum parathyroid hormone (PTH) was not measured, which prevented definitive confirmation of hypomagnesemia‐induced functional hypoparathyroidism as the mechanism of hypocalcemia. Finally, a minor salivary gland biopsy was not performed; had it been positive, it would have provided additional diagnostic confirmation under the 2016 ACR/EULAR criteria.

## Author Contributions

**Premendra Vimal:** conceptualization, supervision, validation. **Dhiraj Chaurasia:** conceptualization, data curation, investigation, writing – original draft, writing – review and editing. **Dipesh Jha:** writing – original draft, writing – review and editing. **Raj Adhikari:** data curation, writing – original draft, writing – review and editing.

## Funding

The authors have nothing to report.

## Ethics Statement

Our institution does not require ethical approval for reporting individual cases or case series.

## Consent

Written informed consent was obtained from the patient to publish this report in accordance with the journal's patient consent policy.

## Conflicts of Interest

The authors declare no conflicts of interest.

## Data Availability

The data that support the findings of this study are available from the corresponding author upon reasonable request.
